# A Contribution to the Treatment of Gonorrhea with Arhovin

**Published:** 1908-02

**Authors:** 


### A Contribution to the Treatment of Gonorrhea with Arhovin

M. Weinberg, Vienna, Austria (*Wiener medicinische Presse,* No. 44 1907), says that since he and Brings, some three years ago, jointly published their experiences with arhovin, he has made continued use of the remedy and the favorable verdict which he now again renders therefore carries with it especial weight.

His observations signally demonstrate the fact that it is certain in effectiveness and, moreover, has the merit of never causing irritative reaction on the part of any organ,—a claim which cannot be made for any of the balsams. For even new and improved balsamics are not altogether free from disagreeable actions on the stomach, intestines and kidneys. On the other hand, not one such instance is reported in the entire literature of arhovin, despite the large number of recorded cases. This fact is of such importance that, even if it were only equal in efficacy to the other medicaments, arhovin is far preferable.

Weinberg also finds arhovin possessed of most remarkable analgesic properties—a fact which he ascribes to the promptness with which it is absorbed. He always noted that even severe pain ceased within two days after its administration, and often on the same day. The astonishing regularity of this effect can only be explained on the ground that arhovin, which is rapidly eliminated, anesthetises the genitourinary organs and arrests the inflammatory processes. Weinberg places the dose somewhat above the average, giving in severe cases ten of the capsules at four grains daily. He attributes his excellent results partly to this increased dosage, from which he never saw untoward effects.

